# Lipid bilayer properties govern substrate engagement and extraction by the AAA+ ATPase Msp1

**DOI:** 10.1016/j.jbc.2025.110614

**Published:** 2025-08-19

**Authors:** Heidi L. Fresenius, Brian Acquaviva, Deepika Gaur, Baylee A. Smith, Matthew L. Wohlever

**Affiliations:** 1 Department of Chemistry & Biochemistry, Previously at University of Toledo, Toledo, Ohio, USA; 2 Department of Cell Biology, University of Pittsburgh, Pittsburgh, Pennsylvania, USA

**Keywords:** ATPase associated with diverse cellular activities (AAA+), membrane protein, proteostasis, mitochondria, lipid bilayer

## Abstract

An essential aspect of protein quality control is enzymatic removal of membrane proteins from the lipid bilayer. Failures in this critical cellular process are associated with neurodegenerative diseases and cancer. Msp1 is a AAA+ (ATPases associated with diverse cellular activities) ATPase that removes mistargeted membrane proteins from the outer mitochondrial membrane. How Msp1 selectively recognizes and extracts substrates within the complex outer mitochondrial membrane ecosystem, and how the lipid bilayer impacts these processes are unknown. Here, we describe the development of a fully defined, rapid, and quantitative extraction assay that retains physiological substrate selectivity. Using this new assay, we systematically modified both the model substrate and the lipid environment to demonstrate that Msp1 can recognize substrates by a hydrophobic mismatch between the substrate transmembrane domain and the lipid bilayer. We further demonstrate that the rate-limiting step in Msp1 activity is extraction of the transmembrane domain from the lipid bilayer. Together, these results provide foundational insights into how the lipid bilayer influences AAA+ mediated membrane protein extraction.

A hallmark of eukaryotic cells is membrane-bound organelles with each organelle characterized by unique protein and lipid compositions (1). As a foundational mechanism for organizing cellular reactions and signaling pathways, the cell devotes significant resources toward establishing and maintaining unique organelle proteomes and lipidomes. Despite the clear importance of targeting membrane proteins to the correct organelle, membrane protein trafficking and insertion pathways are surprisingly error prone and depend heavily on post insertion quality control pathways to correct protein targeting errors (2).

The removal of mislocalized or damaged proteins from the lipid bilayer is typically conducted by AAA+ (ATPase associated with diverse cellular activities) proteins, which are a broad, versatile, and essential family of molecular motors (3, 4). Mutations in AAA+ proteins lead to disrupted protein homeostasis (proteostasis) and are associated with cancer and neurodegenerative diseases (5, 6, 7, 8). AAA+ proteins typically assemble into spiral hexameric rings or lock washers and undergo ATP-dependent movements to translocate a substrate through a central pore (9, 10). Substrate translocation is driven by highly conserved pore loops, which line the central pore and directly contact the substrate (11, 12). The mechanical stress of translocating a substrate through a narrow pore typically results in substrate unfolding (13). Key points of regulation for AAA+ proteins include substrate recognition and access to the central pore (14).

Interestingly, there is only one membrane anchored AAA+ protein in the outer mitochondrial membrane (OMM), Msp1 (15, 16). This protein is responsible for extracting mislocalized substrates, including endoplasmic reticulum (ER) tail anchored (TA) proteins that are mislocalized to the OMM or peroxisomal TA proteins that are in stoichiometric excess of their binding partners (17). Msp1 also helps relieve mitochondrial protein import stress by clearing stalled substrates from the TOM complex (18). ATAD1, the human homolog of Msp1, serves an expanded role in membrane proteostasis by regulating AMPA receptor internalization and selectively removing the proapoptotic protein BIM from the OMM (19, 20). Loss of Msp1/ATAD1 leads to failures in oxidative phosphorylation, impaired fear conditioning, apoptotic priming, and enhanced susceptibility to proteotoxic stress (16, 20, 21). Loss of ATAD1 in mice or humans is lethal (22, 23).

Given the clear health relevance, it is of paramount importance to understand how Msp1 recognizes and extracts substrates. In particular, it is unclear how Msp1 can selectively recognize mislocalized TA proteins from a large excess of properly localized mitochondrial proteins and how these substrates gain access to the central pore. There are currently several competing models in the literature for how Msp1 recognizes substrates (17, 24, 25, 26). These models include recognition of unstructured sequences within the substrate, the reduced thermodynamic stability of a mislocalized substrate, and a hydrophobic patch within the substrate. However, the role of the lipid bilayer in substrate recognition has been largely ignored. Similarly, there are multiple models for how Msp1 engages with substrates (17, 24, 25, 26) (Fig. 1*A*): processive extraction from the N terminus of the substrate, assembly of the hexameric ring around the substrate, or lateral diffusion of the substrate through a seam commonly seen in Cryo-EM structures of AAA+ proteins, including Msp1. These models have profound implications for the mechanism of substrate extraction. For example, the lateral diffusion and ring assembly model would allow substrate translocation to begin in the middle of the substrate, potentially bypassing the need to unfold cytosolic domains.

**Figure 1:**
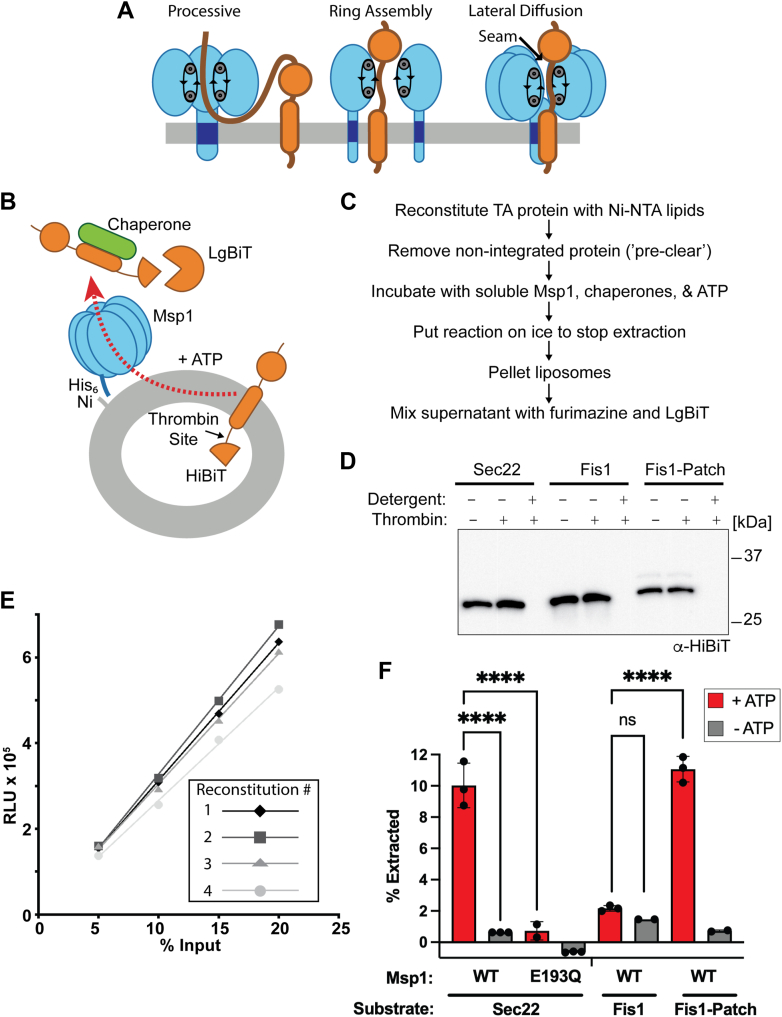
**Validation of the split-luciferase based extraction assay**. *A*, three different models for how Msp1 processes substrates. In the processive model, Msp1 engages with the N terminus of the substrate and processes the entire substrate, resulting in unfolding of any cytosolic domains. In the ring assembly model, the Msp1 hexamer can assemble around a substrate. In the lateral diffusion model, the substrate can laterally diffuse through a seam in the assembled Msp1 hexamer. *B*, diagram for split-luciferase extraction assay. Note that for properly reconstituted liposomes, both the HiBiT and thrombin sites are in the lumen of the proteoliposome. *C*, workflow for split-luciferase assay. *D*, protease protection assay shows that substrates are properly oriented. Anti-HiBiT western blot of precleared liposomes shows minimal loss of signal upon addition of thrombin protease, but complete loss of signal with both thrombin and detergent. *E*, standard curves used to calculate percent substrate extracted. Four separate reconstitutions of the standard SUMO-Sec22 model substrate in standard liposomes show a linear and reproducible signal. Different concentrations of precleared but unreacted proteoliposomes were mixed with LgBiT and furimazine reagent in detergent, which permeablizes the proteoliposomes. The *X*-axis refers to the percent of precleared material in the standard curve relative to the total amount in the extraction assay. *F*, the split-luciferase extraction assay shows ATP-dependent and physiological substrate extraction activity. The known substrate Sec22 is extracted whereas the native mitochondrial protein Fis1 is not extracted unless a hydrophobic patch is added, which serves as an Msp1 recognition sequence. Error bars show standard deviation for 2 to 3 replicates from two separate reconstitutions. One-way ANOVA with Dunnett's *post hoc* test, ∗∗∗∗*p* < 0.0001, ns, not significant.

Here, we study how Msp1 recognizes and extracts a transmembrane domain (TMD) from the lipid bilayer. Using a redesigned *in vitro* extraction assay, we systematically varied the bilayer thickness of the liposomes and TMD length of the model substrate. Our results indicate that Msp1 identifies extraction substrates through a hydrophobic mismatch between the substrate TMD and the lipid bilayer. We also demonstrate that substrate extraction is modulated by changes in lipid bilayer fluidity, but not by increasing the thermodynamic stability or size of the cytosolic domain. These data support a model wherein removal of the TMD from the lipid bilayer, rather than unfolding of cytosolic domain, is the rate-limiting step in Msp1 activity and strongly suggests that substrates access the central pore *via* lateral diffusion rather than processive translocation. Together, these results demonstrate that the lipid bilayer plays a critical role in protein quality control and provide a detailed model for how Msp1 selectively recognizes and removes mislocalized membrane proteins from the far more abundant OMM resident proteins.

## Results

### Optimization of an *in vitro* assay for Msp1 extraction activity

We previously used a reconstituted system to demonstrate that Msp1 is sufficient to recognize and extract a TA protein from a fully defined lipid bilayer (27, 28). Importantly, this reconstituted system showed physiological substrate selectivity as it specifically removed ER-TA proteins, but not the mitochondrial TA protein Fis1 (20). The details of the assay are presented in Figure 1, *A* and *B*. Briefly, this assay involves reconstitution of a model substrate into liposomes followed by a preclearing step to remove nonintegrated substrates. Extracted substrates are captured by the chaperones calmodulin and small glutamine-rich tetratricopeptide repeat-containing protein alpha (SGTA), which recognize the substrate TMD (29, 30, 31). Substrate extraction is monitored by a chaperone immunoprecipitation and western blot against epitopes on the model substrate.

To observe potentially subtle differences in Msp1 activity, we redesigned the extraction assay to eliminate the semi-quantitative and time-consuming western blots. The new assay quantifies Msp1 extraction activity with the NanoLuc split luciferase system (32, 33). We cloned the 11-residue HiBiT sequence onto the C terminus of our substrate, which is sequestered in the lumen of the liposome after reconstitution (Fig. 1*B*).

After reconstitution of substrate into preformed liposomes, the TA protein chaperones SGTA and calmodulin are added to remove any nonintegrated substrate by immunoprecipitation (Fig. 1*C*). After this preclearing step, proper substrate orientation was validated by a protease protection assay that detects the HiBiT peptide. All model substrates contain a thrombin protease site between the TMD and the HiBiT tag (Fig. 1*B*). If the substrate is properly oriented with the thrombin cleavage site sequestered in the lumen of the liposome, then no substrate cleavage will be observed because thrombin cannot cross the lipid bilayer. Addition of the detergent Triton X-100 solubilizes the liposome, allowing thrombin to access the cleavage site. Thrombin cleavage results in HiBiT peptide that is not resolvable by SDS PAGE/western blot. Addition of the thrombin protease led to a minimal loss of signal, whereas addition of both thrombin and the detergent Triton X-100 led to a complete loss of signal, demonstrating that the vast majority of substrate is properly oriented (Fig. 1*D*).

The precleared liposomes not used in the protease protection assay were mixed with Msp1 and fresh SGTA and calmodulin, which were added at this step to capture substrates extracted by Msp1. ATP was then added to initiate the reaction. The assay can be performed with a high concentration of ATP or physiological concentration of ATP with an ATP regeneration system with only modest differences in activity (Fig. S1*C*).

After a 30-min incubation at 30 °C, Msp1 activity is inhibited by chilling the reaction on ice (28) and then liposomes are pelleted by ultracentrifugation. Pelleting the liposomes is necessary to remove the small amount of substrate that reconstituted with the HiBiT tag outside the liposome. We observed that the addition of an inert, His_6_-tagged protein to ultracentrifugation reduces background signal, likely through enhanced liposome pelleting (Fig. S1*D*). It is important to note that the inert, His_6_-tagged protein is added after the extraction assay is stopped by chilling the reaction on ice and is therefore not expected to have any effect on substrate extraction. Repeating the assay with different His-tagged proteins as a pelleting aid had a modest effect on the total amount of extraction activity but was reproducible internally (Fig. 1, *E* and *F*). We chose to use pBS_008 as our inert protein for pelleting across all assays (Table 1).

**Table 1:** Plasmid used in this study

Plasmid	Description	Reference
p299_DG	His_6_-3C-Δ1-32 Msp1	This study
pHF002	His_6_-TEV-Δ1-32 Msp1	This study
pHF003	His_6_-TEV-Δ1-32 Msp1 (E193Q)	This study
pHF027	GST-3C-SGTA	This study
pHF050	GST-3C-Calmodulin	This study
p048_DG	His_6_-3C-LgBiT	This study
pHF122	His_6_-3C-SUMO-Sec22 TMD-thrombin-HiBiT	This study
p448_DG	His_6_-3C-FLAG-SUMO-SUMO-Sec22 TMD-thrombin-HiBiT	This study
p226_DG	His_6_-3C-DHFR-Sec22 TMD-thrombin-HiBiT	This study
pArch_111	His_6_-3C-SUMO-Fis1 TMD-thrombin-HiBiT	This study
pArch_112	His_6_-3C-SUMO-hydrophobic patch-Fis1 TMD-thrombin-HiBiT	This study
pArch_132	His_6_-3C-SUMO-L16 TMD-thrombin-HiBiT	This study
pArch_133	His_6_-3C-SUMO-L18 TMD-thrombin-HiBiT	This study
pArch_134	His_6_-3C-SUMO-L19 TMD-thrombin-HiBiT	This study
pArch_142	His_6_-3C-SUMO-L20 TMD-thrombin-HiBiT	This study
pArch_143	His_6_-3C-SUMO-L22 TMD-thrombin-HiBiT	This study
pArch_144	His_6_-3C-SUMO-L24 TMD-thrombin-HiBiT	This study
pBS_008	His_6_-MBP-Ubiquitin-mNG2	This study
p397_DG	His_10_-3C-sfGFP2	Ref (70)
p581_MLW	His_6_-tdTomato-Nanobody	Ref (71)

DHFR, dihydrofolate reductase; TMD, transmembrane domain.

Following centrifugation, the supernatant is mixed with LgBiT and the luciferase substrate furimazine and luminescence is measured on a plate reader. To quantify total extraction activity, we generated a standard curve by mixing dilutions of precleared liposomes with LgBiT, the luciferase reagent furimazine, and detergent, which leads to membrane permeabilization (Fig. 1*E*).

Another major change is that we used soluble Msp1 instead of full-length Msp1. We and others had previously demonstrated that the Msp1 TMD can be completely changed with no effect on Msp1 activity *in vivo* (17, 28). Similarly, we also demonstrated that soluble Msp1 retains robust and physiologically selective extraction activity *in vitro* if it is anchored to liposomes *via* a Ni-His interaction (20). The use of soluble Msp1 dramatically simplifies the reconstitution process as only the substrate protein needs to be reconstituted rather than co-reconstitution of substrate and six copies of Msp1. This allows us to directly compare extraction efficiency between different liposomes as we have eliminated any potential differences in Msp1 reconstitution efficiency due to changes in lipid composition.

We tested substrate extraction with our standard model substrate, SUMO-Sec22, and standard liposome preparation. These liposomes contain a mixture of synthetic and naturally sourced lipids, including phosphatidyl choline, phosphatidyl ethanolamine, phosphatidyl inositol, phosphatidyl serine, and cardiolipin to mimic the outer mitochondrial membrane (34, 35). We observed robust, ATP dependent extraction (Fig. 1*F*). Extraction efficiency is ∼10% which is comparable to previously published results (20, 27, 28). Compared to our previous western blot-based assay we observe reduced signal in our negative controls. Importantly, the new assay design retains physiological substrate selectivity as the mitochondrial TA protein Fis1 is not extracted in our assay (Fig. 1*F*). However, addition of a known Msp1 recognition sequence, the hydrophobic patch from Pex15, leads to Msp1-dependent extraction consistent with previous results (20, 36). We conclude that the new assay has comparable extraction efficiency with our previously validated assay, but is faster, more quantitative, and has improved signal to noise.

With a robust, quantitative, and physiological extraction assay now established, we next used this assay to examine the effects of the substrate TMD and lipid bilayer on Msp1 extraction activity. As proteoliposome reconstitutions are inherently variable, we took several steps to ensure experimental rigor and reproducibility. The extraction data involve at least two separate reconstitutions and up to three technical replicates within each reconstitution. A new standard curve was generated for each new reconstitution. To ensure proper substrate reconstitution, a protease protection assay and a negative control (no ATP or ATPase deficient E193Q Msp1 mutant) was included in every assay.

### Msp1 extraction activity correlates with a hydrophobic mismatch between the substrate TMD and lipid bilayer

An outstanding question in the field is how Msp1 recognizes substrates (24). Our data demonstrate that the hydrophobic patch is sufficient for substrate recognition (Fig. 1*F*). However, the juxtamembrane hydrophobic patch is unique to Pex15 and does not appear to be widely conserved across other Msp1 substrates. We have previously proposed that Msp1 recognizes substrates by a hydrophobic mismatch between the TMD of the mislocalized substrate and the lipid bilayer (24).

**Figure 2:**
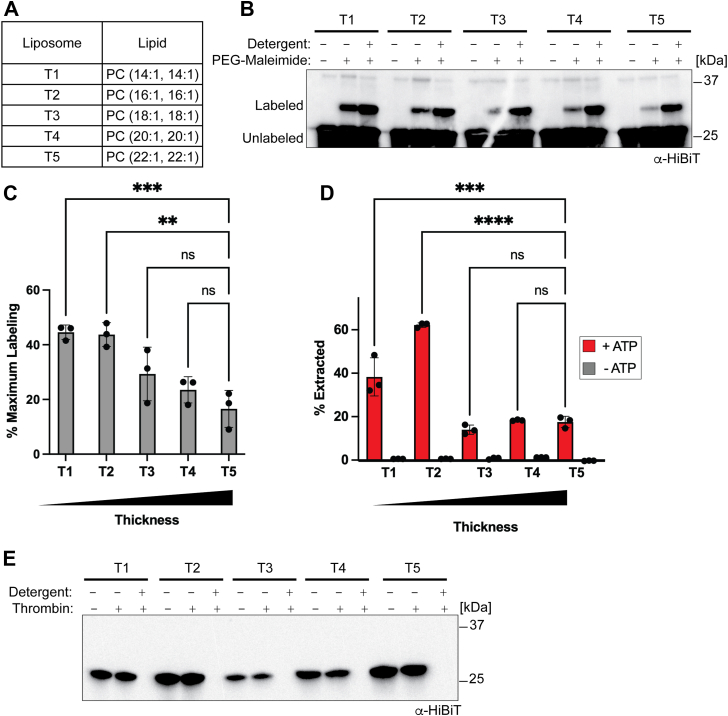
**Msp1 extraction activity correlates with hydrophobic mismatch between the substrate TMD and lipid bilayer.***A*, composition of T-series liposomes. *B*, PEG-maleimide labeling shows increased TMD exposure in thinner liposomes. *C*, quantification of data (*B*) from three separate reconstitutions. Error bars show standard deviation. The amount of labeled material in the + detergent sample is defined as 100% labeling. Error bars show standard deviation for three replicates. One-way ANOVA with Dunnett's *post hoc* test ∗∗∗*p* < 0.001 and ∗∗*p* < 0.01. *D*, extraction of the standard SUMO-Sec22 substrate in T-series liposomes. Msp1 shows more robust extraction activity in thinner liposomes. Error bars show standard deviation for three replicates from two separate reconstitutions. One-way ANOVA with Dunnett's *post hoc* test ∗∗∗∗*p* < 0.0001 and ∗∗∗*p* < 0.001. *E*, protease protection assay of standard SUMO-Sec22 substrate in T-series liposomes. TMD, transmembrane domain.

To test this hypothesis, we first attempted to generate a hydrophobic mismatch by reconstituting the Sec22 model substrate into a series of liposomes with varying chain lengths. To minimize heterogeneity in the system, all liposomes were composed of phosphatidylcholine with both acyl chains containing a single cis double bond. The liposomes also contained 2% of DOGS-NiNTA, which was necessary to anchor soluble Msp1 to the liposomes. For simplicity, we will refer to the thickness liposomes as our T-series liposomes, with T1 referring to the thinnest liposomes (14:1, 14:1) and T5 the thickest liposomes (22:1, 22:1) (Fig. 2*A*).

We first sought to demonstrate increased exposure of TMD residues in a thinner lipid bilayer. To do this, we measured the solvent accessibility of a membrane-adjacent cysteine residue in our model substrate. We added a single cysteine residue immediately to the N-terminus of the Sec22 TMD. We then reconstituted the substrate into our T-series liposomes and reacted the liposomes with PEG_5000_-maleimide. If the cysteine residue is buried in the lipid bilayer, it will be protected from the membrane impermeable crosslinker. Conversely, a hydrophobic mismatch will lead to increased solvent exposure of the cysteine residue, resulting in increased labeling by PEG_5000_-maleimide, which can be easily observed by a size shift on SDS PAGE. As a positive control, the liposomes were solubilized with 1% Triton X-100 during labeling. The overall trend is a decrease in cysteine labeling as the model substrate moves to thicker liposomes, although only T1 and T2 liposomes have a statistically significant difference from T5 liposomes (Fig. 2, *B* and *C*).

Having demonstrated that thinner bilayers lead to increased solvent exposure of the TMD, we next asked if substrate extraction is enhanced in thinner bilayers. Consistent with our hypothesis, we observed higher levels of substrate extraction in the thinner liposomes than the thicker liposomes (Fig. 2*D*). Importantly, a protease protection assay again shows that the vast majority of substrate is properly oriented for all substrates (Fig. 2*E*). Therefore, the amount of solvent exposed cysteine is not due to differences in substrate orientation.

Unlike the linear drop in PEG-maleimide labeling, the drop in substrate extraction appeared to follow a step function with robust extraction in the T1 and T2 liposomes. Interestingly, the extraction assay results mirror the maleimide labeling assay, with extraction from the T1 and T2 liposomes being the only samples that are statistically different from the T5 liposomes. The extraction in the T3-T5 liposomes is comparable to the mitochondrial liposomes (Fig. 1*F*), which is reasonable given that the acyl chain lengths in the T3 to T5 liposomes are largely similar to the complex mixture of lipids used in the mitochondrial liposomes. We conclude that there is a strong positive correlation between hydrophobic mismatch of the substrate TMD with the lipid bilayer and Msp1 extraction activity.

### Substrates with a longer TMD show enhanced extraction activity

Many factors govern the formation of a hydrophobic mismatch between a TMD and a lipid bilayer, including TMD length and secondary structure, bilayer thickness, membrane curvature, local membrane packing defects, and the tilt of a TMD relative to the bilayer plane (37, 38, 39, 40, 41, 42). In addition to enhanced hydrophobic mismatch in thinner bilayers, we hypothesized that the enhanced substrate extraction in the thinner bilayers could also be due to a lower thermodynamic barrier for substrate extraction (43). Indeed, previous studies have shown that as the acyl chain length increases, the order of the lipid molecules increases (44).

To determine whether the enhanced extraction in the thinner liposomes is due to increased hydrophobic mismatch or a lower thermodynamic barrier, we generated a hydrophobic mismatch by increasing the length of the TMD while holding the lipid bilayer composition constant. Increasing the length of the TMD should increase the stability of the substrate in the lipid bilayer, thereby increasing the thermodynamic barrier for substrate extraction (45, 46). If substrates with a longer TMD are extracted more robustly, it will be due to enhanced recognition by Msp1 rather than a lower thermodynamic barrier for extraction.

**Figure 3:**
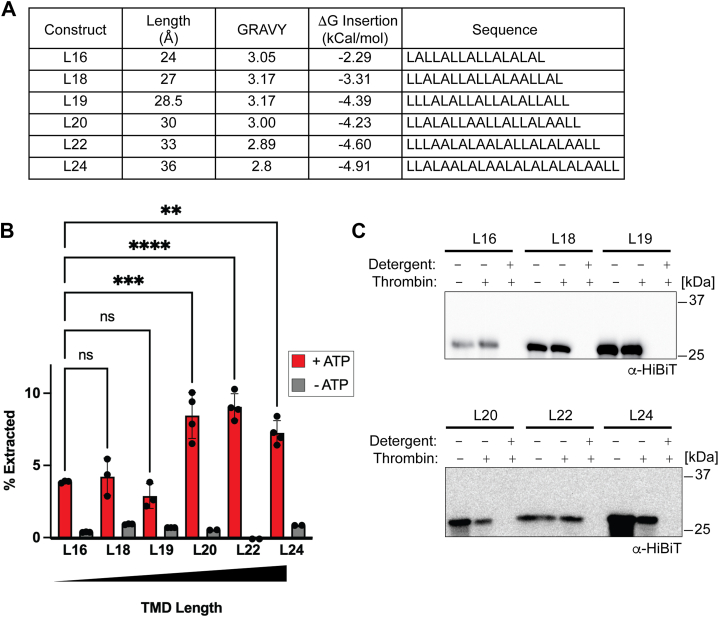
**Design and extraction of L-series substrates**. *A*, design of L-series substrates. The length of the TMD is calculated by assuming a rise of 1.5 Å per residue. GRAVY score was calculated by inserting the TMD sequence into the GRAVY calculator (https://www.gravy-calculator.de/). The ΔG for TMD insertion was calculated using the website (https://dgpred.cbr.su.se/index.php?p=TMpred) (45, 46). TMD sequences for L-series substrates all start and end with leucine. *B*, L-series substrates L20-L24, which are predicted to have a hydrophobic mismatch, exhibit enhanced extraction activity. L-series substrates were reconstituted in standard liposomes. Error bars show standard deviation for 3 to 4 replicates (+ATP) or 2 to 3 replicates (−ATP) from two separate reconstitutions. One-way ANOVA with Dunnett's *post hoc* test ∗∗∗∗*p* < 0.0001, ∗∗∗*p* < 0.001, and ∗∗*p* < 0.01. *C*, protease protection assay of L-series substrates in standard liposomes. TMD, transmembrane domain; GRAVY, grand average of hydrophobicity.

We therefore created a series of artificial substrates with TMD lengths ranging from 16 to 24 residues. For reference, Deep TMHMM predicts that the Sec22 model has a TMD length of 20 residues (47). To prevent Msp1 interaction with specific sequences in the model substrate from potentially biasing our results, we designed artificial TMDs composed solely of leucine and alanine residues (48) (Fig. 3*A*). To standardize the substrates, all TMDs were required to begin and end with leucine, have a grand average of hydrophobicity (GRAVY) score of 3 ± 0.2, and contain 10 to 13 leucine residues (49). We refer to these as our L-series substrates, with L16 having the shortest TMD at 16 residues and L24 having the longest TMD at 24 residues. The only difference between L-series substrates and the Sec22 model substrate is the TMD sequence and length.

We used an online calculator to predict the change in free energy for insertion of the L-series constructs into a model membrane (45, 46) (Fig. 3*A*). As expected, TMD insertion into the bilayer was more favorable with a longer construct. The thermodynamic barrier model predicts that the longer constructs should therefore have higher thermodynamic barriers for removal from the lipid bilayer and lower overall extraction. Conversely, the hydrophobic mismatch model predicts that the longer TMDs should lead to increased hydrophobic mismatch and therefore enhanced substrate extraction.

To distinguish between these two models, the L-series substrates were reconstituted into our standard liposomes and used in our extraction assay. All constructs showed ATP dependent extraction with the longer constructs showing higher levels of extraction than the shorter constructs (Fig. 3*B*). We again observed a step type function rather than a continuous change, with L20, L22, and L24 showing higher extraction than L16, L18, and L19 (Fig. 3*B*). Protease protection assays showed that the substrates were reconstituted in the proper orientation (Fig. 3*C*). Although the change in extraction activity is relatively modest, it is significant and reproducible. The simplest explanation for the modestly enhanced extraction of the L20-L24 constructs is that a hydrophobic mismatch leads to enhanced substrate recognition, which is partially counteracted by increased thermodynamic stability of the substrate in the lipid bilayer.

### Variation of both substrate length and bilayer thickness supports the hydrophobic mismatch model

Our standard liposomes contain a complex mixture of natural and synthetic lipids which make it difficult to precisely predict the thickness of the hydrophobic core of the lipid bilayer. We also observed that a large discrepancy between TMD length and bilayer thickness led to low reconstitution efficiency. We hypothesized that these complications led to the nonlinear trends in our extraction assays. To test this hypothesis, we reconstituted the L-series substrates into the T-series liposomes, thereby gaining full experimental control over both TMD length and bilayer thickness.

**Figure 4:**
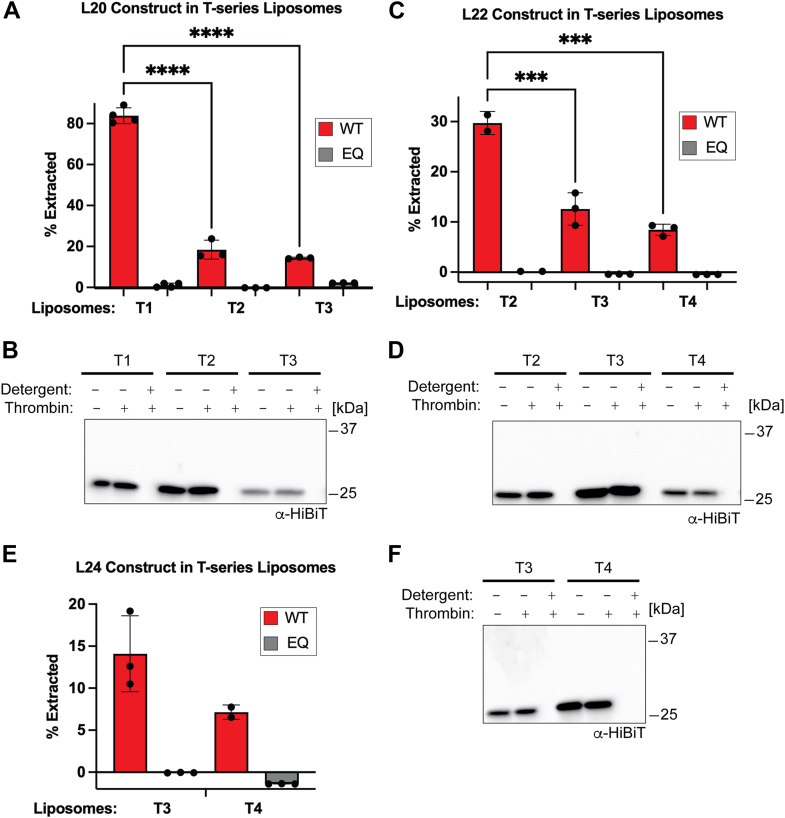
**Extraction of L-series substrates in T-series liposomes**. *A*, extraction of the L20 substrate is most robust when there is a predicted hydrophobic mismatch. L20 substrate was reconstituted in liposomes T1-T3. Based on the length of the TMD and thickness of hydrophobic core of the lipid bilayer (44), there is a predicted hydrophobic mismatch in T1, a possible mismatch in T2, and no predicted mismatch in T3. Error bars show standard deviation for 3 to 4 replicates from two separate reconstitutions. One-way ANOVA with Dunnett's *post hoc* test ∗∗∗∗*p* < 0.0001. *B*, protease protection assay for proteoliposomes in (*A***)**. *C*, similar to (*A*), L22 substrate was reconstituted in liposomes T2-T4. There is a predicted hydrophobic mismatch in T2, a possible mismatch in T3, and no predicted mismatch in T4. Error bars show standard deviation for 2 to 3 replicates from two separate reconstitutions. One-way ANOVA with Dunnett's *post hoc* test ∗∗∗*p* < 0.001. *D*, protease protection assay for proteoliposomes in (*C*). *E*, similar to (*A*), L24 substrate was reconstituted in liposomes T3 and T4. There is a predicted hydrophobic mismatch in T3 and no predicted mismatch in T4. Error bars show standard deviation for 2 to 3 replicates from two separate reconstitutions. *F*, protease protection assay for proteoliposomes in (*E*). TMD, transmembrane domain.

We reconstituted the L20, L22, and L24 substrates into the T-series liposomes that most closely matched the predicted TMD length (44). For the L20 substrate, this involved reconstitution into T1, T2, and T3 liposomes. Based on the length of the L20 TMD and the thickness of the T-series liposomes, there should be a hydrophobic mismatch in T1, a possible mismatch in T2, and no mismatch in T3 liposomes. Consistent with this model, we observed the most robust extraction in T1 proteoliposomes and the weakest extraction in the T3 liposomes (Fig. 4*A*). There are no significant differences in extraction between T2 and T3 liposomes.

We hypothesized that by increasing the length of the substrate TMD, we could generate a hydrophobic mismatch in liposomes with a thicker hydrophobic core. Therefore, we reconstituted the L22 model substrate in the T2-T4 liposomes. Consistent with our hypothesis, we observed robust extraction in T2 liposomes and with only modest differences between T3 and T4 liposomes (Fig. 4*C*). A similar trend was observed with L24 in T3 and T4 liposomes (Fig. 4*E*). The protease protection assay shows that the substrates are properly oriented (Fig. 4, *B*, *D*, and *F*). We conclude that a hydrophobic mismatch between a substrate TMD and the lipid bilayer is a major driver of Msp1 substrate selectivity.

### Substrate extraction is more robust in bilayers with enhanced fluidity

Having established that a hydrophobic mismatch between the substrate TMD and the lipid bilayer is a major determinant of Msp1-mediated extraction, we next sought to define the rate-limiting step in substrate extraction. Previous studies with soluble Msp1 working on soluble substrates concluded that Msp1 is a processive unfoldase that recognizes long, unstructured tails at the end of a substrate (26). This model predicts that Msp1 must first unfold cytosolic domains on a substrate before removing the TMD from the bilayer. In such a model, there are two major thermodynamic barriers that could serve as the rate-limiting step in the reaction, (1) unfolding of the cytosolic domain or (2) extraction of the TMD from the lipid bilayer.

To test if TMD removal from the lipid bilayer is the rate-limiting step in substrate extraction, we reconstituted our standard Sec22 model substrate into liposomes with increasing levels of unsaturated acyl chains. If unfolding of the cytosolic domain is the rate-limiting step in substrate extraction, then increasing the fluidity of the lipid bilayer should have no effect on substrate extraction. Conversely, if the rate-limiting step is removal of the TMD from the bilayer, we expect to see enhanced extraction activity in the more fluid membrane.

**Figure 5:**
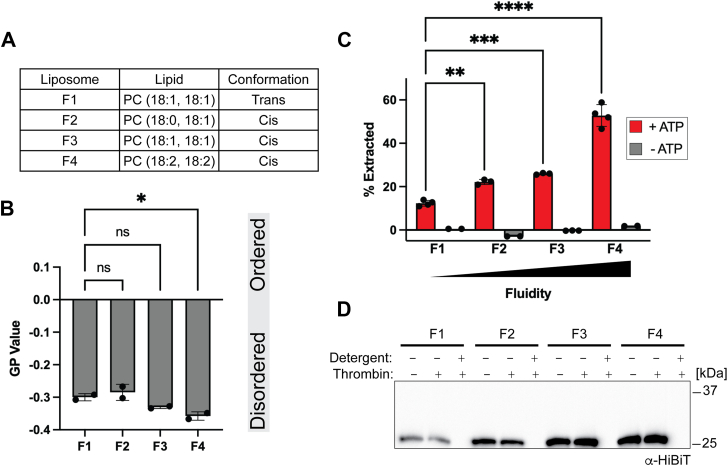
**Msp1 extraction activity is enhanced with increasing membrane fluidity**. *A*, composition of fluidity series liposomes. *B*, lipid packing of F-series liposomes measured by C-laurdan spectroscopic measurements. Generalized polarization (GP) values range from +1 (most ordered) to −1 (most disordered). Error bars show standard deviation for two replicates from two separate reconstitutions. One-way ANOVA with Dunnett's *post hoc* test ∗ *p* < 0.05. *C*, extraction of standard SUMO-Sec22 substrate in F-series liposomes. Error bars show standard deviation for 3 to 4 replicates (+ATP) and 2 to 3 replicates (−ATP) from two separate reconstitutions. One-way ANOVA with Dunnett's *post hoc* test ∗∗∗∗*p* < 0.0001, ∗∗∗*p* < 0.001, and ∗∗*p* < 0.01. *D*, protease protection assay for proteoliposomes in (*C*).

To minimize heterogeneity in the system, all liposomes were composed of phosphatidylcholine with an acyl chain length of 18 (Fig. 5*A*). We will refer to the fluidity liposomes as our F-series liposomes, with F1 referring to the least fluid and F4 the most fluid. All liposomes were prepared in an identical manner and extruded through a 100 nm pore size. The molecular lipid packing densities were determined by using the fluorescent reporter C-laurdan. The generalized polarization (GP) values align with previously published values and follow the expected trend (50) (Fig. 5*B*). Consistent with our hypothesis that TMD extraction is a rate-limiting step, we observed an increase in substrate extraction with our most fluid liposomes (Fig. 5*C*). A protease protection assay again shows that the substrates are properly oriented (Fig. 5*D*).

### Changing the stability of the cytosolic domain has no effect on substrate extraction

**Figure 6:**
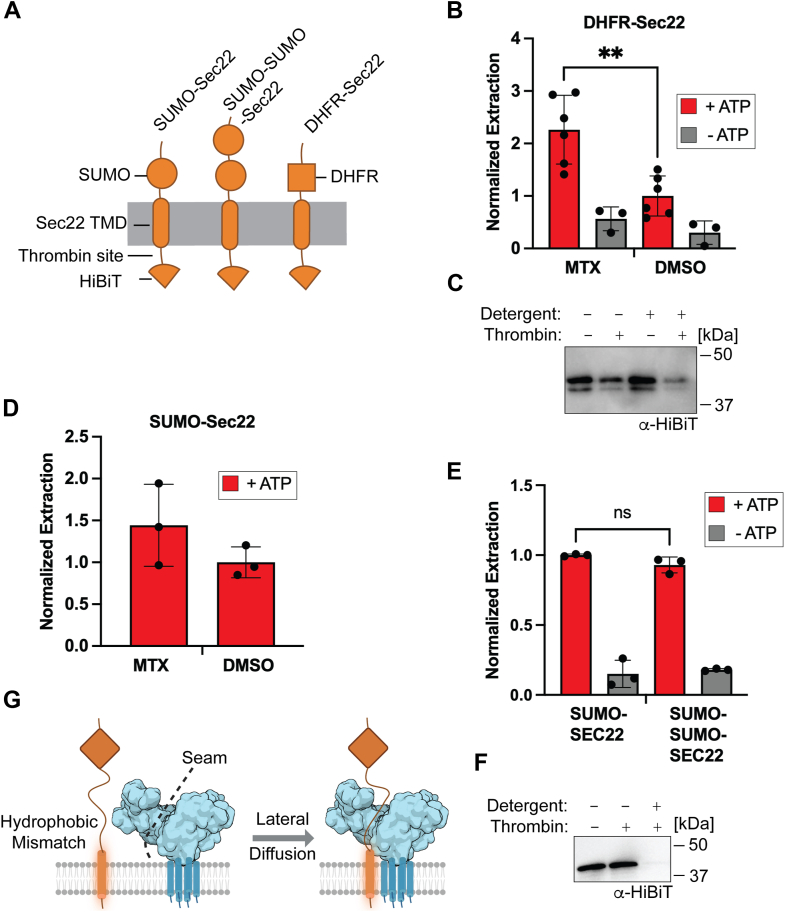
**The rate-limiting step in Msp1 activity is likely removal of the substrate TMD from the lipid bilayer**. *A*, diagram of SUMO-Sec22, SUMO-SUMO-Sec22, and DHFR-Sec22 model substrates. Note that the only difference is in the cytosolic domain. *B*, addition of methotrexate (MTX) leads to an increase in extraction of the DHFR-Sec22 model substrate. Activity normalized such that DMSO + ATP is 1.0. Error bars show standard deviation for 3 (−ATP) to 6 (+ATP) replicates from two separate reconstitutions. Unpaired, two-tailed *t* test ∗∗*p* < 0.01. *C*, protease protection assay for the DHFR-Sec22 substrate in standard liposomes. *D*, addition of methotrexate leads to an increase in extraction of SUMO-Sec22 model substrate. Activity normalized such that DMSO + ATP is 1.0. Note that both the DMSO and methotrexate samples have ATP added. Error bars show standard deviation for three replicates from two separate reconstitutions. *E*, extraction of the SUMO-Sec22 and SUMO-SUMO-Sec22 model substrates are nearly identical. Activity normalized such that SUMO-Sec22 + ATP is 1.0. Error bars show standard deviation for three replicates from two separate reconstitutions. No significant difference between +ATP samples by unpaired, two-tailed *t* test. *F*, protease protection assay with SUMO-SUMO-Sec22 model substrate. *G*, diagram of the lateral diffusion model into the Msp1 hexamer. Mislocalized ER-TA proteins have a hydrophobic mismatch with the OMM, leading to interaction between the solvent exposed TMD and hydrophobic residues in the seam of the Msp1 hexamer. The substrate enters the central pore of Msp1 by laterally diffusing through the seam, potentially bypassing the need for unfolding of cytosolic domains. OMM, outer mitochondrial membrane; DMSO, dimethyl sulfoxide; ER, endoplasmic reticulum; DHFR, dihydrofolate reductase; TA, tail anchored; TMD, transmembrane domain.

If TMD removal from the lipid bilayer is the rate-limiting step in substrate extraction, then changing the size or thermodynamic stability of the cytosolic domain should have no effect on Msp1 extraction activity. To test this hypothesis, we replaced the SUMO domain in our model substrate with dihydrofolate reductase (DHFR) (Fig. 6*A*). Addition of the inhibitor methotrexate increases the resistance of DHFR to mechanical unfolding by AAA proteins (51). Conversely, methotrexate has been shown to interact with phospholipids to increase membrane fluidity (52, 53). If unfolding of the cytosolic domain is the rate-limiting step, then addition of methotrexate should decrease substrate extraction. Conversely, if extraction from the lipid bilayer is the rate-limiting step, then addition of methotrexate should increase substrate extraction.

We reconstituted the DHFR-Sec22 model substrate into our standard liposomes and performed the extraction assay in the presence of methotrexate or dimethyl sulfoxide (DMSO) as a negative control. We observed an increase in substrate extraction upon addition of methotrexate (Fig. 6*B*), consistent with TMD extraction being the rate-limiting step. A protease protection assay shows that the substrate is mostly in the correct orientation (Fig. 6*C*). To confirm that the enhanced substrate extraction observed upon addition of methotrexate is a general phenomenon, we repeated the extraction assay with the standard SUMO-Sec22 model substrate, which should not interact with methotrexate. We again observed a stimulation of substrate extraction upon addition of methotrexate (Fig. 6*D*).

There are different models for how Msp1 engages with substrates (Fig. 1*A*). The processive model requires unfolding of the cytosolic domains whereas the ring-assembly and lateral diffusion models allow for Msp1 to initially engage with the middle of the substrate, potentially bypassing the need to unfold cytosolic domains. To differentiate between these models, we generated the SUMO-SUMO-Sec22 model substrate, which has two consecutive SUMO domains facing the cytosol. Because the SUMO domain is a thermodynamically stable substrate with a higher T_M_ for thermal denaturation than DHFR (54, 55), we hypothesized that if the processive model is correct, then doubling the number of cytosolic domains that require unfolding and translocation would lead to a decrease in substrate extraction.

Contrary to the prediction of the processive model, there was no significant difference in the extraction of the SUMO-Sec22 and SUMO-SUMO-Sec22 model substrates (Fig. 6*E*). Reconstitution of the SUMO-SUMO-Sec22 model substrate showed the expected orientation (Fig. 6*F*). As changing the size and thermodynamic stability of the soluble domain has no effect on substrate extraction whereas increasing bilayer fluidity enhances substrate extraction, we conclude that the rate-limiting step in Msp1 activity is extraction of the TMD from the lipid bilayer and that Msp1 is unlikely to utilize a processive model of substrate extraction.

## Discussion

An essential aspect of membrane proteostasis is the removal of substrates from the membrane. However, our mechanistic understanding of this process, particularly the role of the lipid bilayer, is incomplete. To address this knowledge gap, we developed a rapid and quantitative assay to measure Msp1-mediated substrate extraction from a lipid bilayer. We demonstrated that this assay has comparable extraction efficiency to our previously published assay but with the added benefits of greater quantitation, faster turnaround time, and reduced background signal. Importantly, this system retains physiological substrate selectivity. Using this fully defined system, we systematically varied features of our model substrates and the lipid bilayer to understand the molecular basis for how Msp1 recognizes and removes substrates from the lipid bilayer. We demonstrate that increasing the hydrophobic mismatch between the substrate TMD and the lipid bilayer leads to more robust extraction, suggesting that Msp1 recognizes substrates *via* a hydrophobic mismatch. Furthermore, we demonstrate that increasing the fluidity of the membrane enhances substrate extraction whereas changing the size and thermodynamic stability of the cytosolic domain has no effect on substrate extraction. We conclude that the rate-limiting step in substrate extraction is removal of the TMD from the lipid bilayer.

Previous work had led to the proposal of several different models for how Msp1 recognizes and extracts substrates. Weir *et al.* demonstrated that Msp1 extracts Pex15 from the peroxisome when it is in stoichiometric excess of its binding partner, Pex3 (17). This led to a model that orphaned substrates are less thermodynamically stable than a mature complex and/or expose an Msp1 recognition motif. However, subsequent studies demonstrated that Pex3 can directly inhibit Msp1 activity (26). Using a soluble Msp1 construct that was artificially hexamerized by fusion to the PAN N-domain, Castanzo *et al.* demonstrated that Msp1 recognizes long, unstructured polypeptides (26). However, the rate of substrate processing was quite slow, with a V_Max_ of 0.1 substrates hexamer^−1^ min^−1^. It should also be noted that these studies used soluble (ΔTMD) Msp1 and soluble substrates in the absence of membranes, so the physiological significance is unclear. Both of these models suggest that substrate thermodynamic stability plays a major role in regulating Msp1 activity.

Li *et al.* used photo-crosslinking to demonstrate that a juxtamembrane hydrophobic patch is critical for Msp1-Pex15 interactions (36). Addition of this hydrophobic patch to the mitochondrial TA protein Fis1 is sufficient to convert it into an Msp1 substrate. This is a particularly appealing model as recent cryo-EM structures of *Chaetomium thermophilum* Msp1 and human ATAD1 show an exposed hydrophobic pocket within the N domain that could provide a membrane adjacent binding site for such a hydrophobic patch (56, 57). However, few Msp1 substrates contain similar hydrophobic patches, raising questions about the generality of a hydrophobic patch.

We propose that a hydrophobic mismatch between the substrate TMD and the lipid bilayer is the primary mechanism for Msp1 recognition. This model is particularly enticing as the predominant substrates for Msp1, mislocalized ER-TA proteins, tend to have longer and more hydrophobic TMDs than mitochondrial TA proteins (58). Therefore, mislocalized ER-TA proteins will likely have a hydrophobic mismatch with the OMM, leading to exposed hydrophobic residues that drive Msp1 engagement.

Although our data are consistent with Msp1 recognizing substrates *via* a hydrophobic mismatch, there are likely additional factors that modulate substrate extraction. For example, the results with the T-series and F-series liposomes are reminiscent of a step function rather than a linear increase. Previous studies have shown that a hydrophobic mismatch can lead to packing defects in the lipid bilayer and/or tilting of the TMD (37, 38, 39, 40, 41, 42). Furthermore, acyl chain length and the presence of polyunsaturated fatty acids affect spontaneous lipid curvature, which may in turn affect Msp1 activity (59, 60). Future work should focus on clearly defining how the hydrophobic mismatch affects the lipid bilayer and substrate TMD, how Msp1 recognizes a hydrophobic mismatch, where it occurs in the bilayer, and the influence of lipid headgroups on substrate recognition.

If the hydrophobic mismatch is the predominant means of substrate recognition, then Msp1 is likely engaging with the middle of the substrate rather than the terminus. This raises the question of how a membrane anchored substrate can gain access to the axial pore of the membrane anchored Msp1 ring. As previously proposed, there are multiple potential models that overcome this geometric problem (24, 25). First, a substrate could laterally diffuse into the axial pore through a gap in the Msp1 ring. Second, the Msp1 ring could assemble around a substrate. The ring assembly model is supported by biochemical data showing that WT Msp1 does not form a constitutive hexamer *in vitro* or *in vivo* (28, 36). The lateral diffusion model is supported by cryo-EM structures of ATPase inactive Msp1 hexamers in a lock-washer conformation with a seam that could support lateral diffusion of the substrate into the axial pore (56, 57).

A particularly attractive feature of the lateral diffusion model is that the cryo-EM structure of Msp1 shows an exposed hydrophobic groove in the N domain of the Msp1 subunit that forms part of the seam in the lock washer (57). Cross-linking studies have shown that this hydrophobic groove interacts with substrates (36). As the N domain is exposed to the lipid bilayer, this provides an obvious mechanism for simultaneously recognizing a hydrophobic mismatch and facilitating lateral diffusion of the substrate into the axial pore (Fig. 6*G*). Rigorously distinguishingly between these models should be a high priority.

Although our results demonstrate a prominent role for a hydrophobic mismatch in substrate recognition, they do not exclude the possibility of processive substrate unfolding driven by the Msp1 pore loops directly engaging with long, unstructured regions on the substrate terminus. Indeed, many AAA+ proteins have bipartite substrate recognition motifs, with long-unstructured regions showing nonsequence specific engagement with the pore loops (61, 62).

Our model also raises the possibility that Msp1 activity is regulated by changes in lipid composition of the OMM. Indeed, previous lipidomic work has shown that the OMM is one of the most fluid membranes in the cell, which should support robust Msp1 activity (63). Mitochondria also play a major role in lipid metabolism, with ER–mitochondria contact sites facilitating the exchange of lipids between the two membranes (64). Interestingly, Msp1 appears to preferentially engage with substrates at ER–mitochondria contact sites (65), suggesting that Msp1 may be particularly sensitive to changes in lipid metabolism.

Another possibility is that Msp1 activity is regulated by scramblases. Previous studies demonstrated that scramblases lead to lipid bilayer thinning, and PLSCR3 has been newly identified as an OMM scramblase (66, 67). This could potentially increase Msp1 activity by enhancing hydrophobic mismatch or reducing the energetic barrier for TMD extraction. Interestingly, the AAA+ protease FtsH was recently shown to have scramblase activity (68). Our use of soluble Msp1 precludes our ability to test if Msp1 also has scramblase activity. However, multiple groups generated Msp1 TMD chimeras and observed no defect in Msp1 extraction activity *in vivo* (17, 28). Therefore, if the Msp1 TMDs have scramblase activity, the effect is either independent of the TMD sequences or outside the resolution of the assays. Future studies combining high resolution lipidomic work with *in vivo* Msp1 activity measurements (69) will be important for enhancing our understanding of how changes in lipid composition regulate Msp1 activity.

In conclusion, we have developed a new assay to monitor the extraction of membrane proteins from a lipid bilayer by the AAA+ motor protein Msp1. Using this fully defined system, we systematically probed the role of the membrane composition and substrate TMD on Msp1 activity. We discovered that Msp1 recognizes substrates by a hydrophobic mismatch between the substrate TMD and the lipid bilayer. We also demonstrated that the rate-limiting step in substrate extraction is removal of the TMD from the lipid bilayer. Together, this work provides foundational insights into how Msp1 and other membrane extractases perform the essential function of removing membrane proteins from a lipid bilayer.

## Experimental procedures

### Soluble protein purification

#### Δ1-32 Msp1

Plasmids were transformed into *Escherichia coli* BL21(DE3) or LOBSTR cells containing a pRIL plasmid and grown in terrific broth at 37 °C until an *A*_600_ of 0.6 to 1.0. Cultures were induced with 0.25 mM IPTG and grown at room temperature (RT) for an additional 3 to 4 h. Cells were harvested by centrifugation, and resuspended in Msp1 lysis buffer (20 mM Tris pH 7.5, 200 mM KAc, 20 mM imidazole, 0.01 mM EDTA, and 1 mM DTT) supplemented with 0.05 mg/ml lysozyme (Sigma-Aldrich), 1 mM phenylmethylsulfonyl fluoride (PMSF) and 500 U of universal nuclease (Pierce), and lysed by sonication. The supernatant was isolated by centrifugation for 30 min at 4 °C at 18,500*g* and purified by Ni-NTA affinity chromatography (Pierce) on a gravity column. Ni-NTA resin was washed with 20 column volumes (CV) of Msp1 lysis buffer and then 10 CV of wash buffer (Msp1 lysis buffer with 30 mM imidazole) before elution with lysis buffer supplemented with 250 mM imidazole.

The protein was further purified by size-exclusion chromatography (SEC) (Superdex 200 Increase 10/300 Gl, GE Healthcare) in 20 mM Tris pH 7.5, 200 mM KAc, and 1 mM DTT. Peak fractions were pooled, concentrated to 5 to 15 mg/ml in a 30 kDa molecular weight cutoff (MWCO) Amicon Ultra centrifugal filter (Pierce) and aliquots were flash-frozen in liquid nitrogen and stored at −80 °C. Protein concentrations were determined by A_280_ using a calculated extinction coefficient (Expasy).

#### SGTA and calmodulin

GST-SGTA and GST-calmodulin were expressed as described above for soluble Msp1 constructs. Cells were harvested by centrifugation and resuspended in SGTA lysis buffer (50 mM Hepes pH 7.5, 150 mM NaCl, 0.01 mM EDTA, 1 mM DTT, and 10% glycerol) supplemented with 0.05 mg/ml lysozyme (Sigma-Aldrich), 1 mM PMSF, and 500 U of universal nuclease (Pierce), and lysed by sonication. The supernatant was isolated by centrifugation for 30 min at 4 °C at 18,500*g* and purified by glutathione affinity chromatography (Thermo Fisher Scientific) on a gravity column. Resin was washed with 20 CV of SGTA lysis buffer and then eluted with three CV of SGTA lysis buffer supplemented with 10 mM reduced glutathione. The protein was further purified by SEC (Superdex 200 Increase 10/300 GL, GE Healthcare) in 20 mM Tris pH 7.5, 100 mM NaCl, and 0.1 mM TCEP. Peak fractions were pooled, concentrated to 10 to 20 mg/ml in a 30 kDa MWCO Spin Concentrator (Pierce) and aliquots were flash-frozen in liquid nitrogen and stored at −80 °C. Protein concentrations were determined by *A*_280_ using a calculated extinction coefficient (Expasy).

#### LgBiT and MBP-Ubiquitin

LgBiT and MBP-Ubiquitin were expressed as described above for soluble Msp1 constructs. Cells were harvested by centrifugation and resuspended in lysis buffer (20 Tris 7.5, 200 NaCl, 1 DTT, 0.01 EDTA, and 20 mM imidazole) supplemented with 0.05 mg/ml lysozyme (Sigma-Aldrich), 1 mM PMSF, and 500 U of universal nuclease (Pierce), and lysed by sonication. The supernatant was isolated by centrifugation for 30 min at 4 °C at 18,500*g* and purified by Ni-NTA affinity chromatography (Pierce) on a gravity column. Ni-NTA resin was washed with 20 CV of lysis buffer and then 10 CV of wash buffer (20 Tris 7.5, 200 NaCl, 1 DTT, 0.01 EDTA, and 50 mM imidazole) before elution with lysis buffer supplemented with 250 mM imidazole. The protein was further purified by SEC (Superdex 200 Increase 10/300 GL, GE Healthcare) in 20 mM Tries pH 7.5, 200 mM NaCl, and 1 mM DTT. Peak fractions were pooled, concentrated to 5 to 15 mg/ml in a 30 kDa MWCO Amicon Ultra centrifugal filter (Pierce) and aliquots were flash-frozen in liquid nitrogen and stored at −80 °C. Protein concentrations were determined by *A*_280_ using a calculated extinction coefficient (Expasy).

#### P581_MW

P581_MW was expressed as described above for soluble Msp1 constructs. Cells were harvested by centrifugation and resuspended in lysis buffer (50 Tris 7.5, 200 NaCl, 1 DTT, 0.01 EDTA, 20 mM imidazole, and 10% glycerol) supplemented with 0.05 mg/ml lysozyme (Sigma-Aldrich), 1 mM PMSF and 500 U of universal nuclease (Pierce), and lysed by sonication. The supernatant was isolated by centrifugation for 30 min at 4 °C at 18,500*g* and purified by Ni-NTA affinity chromatography (Pierce) on a gravity column. Ni-NTA resin was washed with 20 CV of lysis buffer and then 10 CV of wash buffer (100 Tris 7.5, 500 NaCl, 1 DTT, 0.01 EDTA, 30 mM imidazole, and 10% glycerol) before elution with 100 Tris 7.5, 150 NaCl, 1 DTT, 0.01 EDTA, 200 mM imidazole, 10% glycerol, and 0.1% Tween-20. Elution was concentrated to 15 mg/ml and buffer exchanged into dialysis buffer (20 mM Tris pH 7.5, 200 mM NaCl, and 1 mM DTT) in a 50 kDa MWCO Thermo Ultra centrifugal filter (Pierce). Protein concentrations were determined by A_280_ using a calculated extinction coefficient (Expasy).

#### P397_DG

Expression of purification of p397_DG was performed as previously described (70). Briefly, plasmid was transformed into BL21 (DE3) cells containing a pRIL plasmid and grown in terrific broth at 37 °C until an *A*_600_ of 0.6 to 1.0. Cultures were induced with 0.3 mM IPTG and grown at 16 °C for an additional 16 h. Cells were pelleted by centrifugation and resuspended in lysis buffer (20 mM Tris pH 7.5, 200 mM potassium acetate, 20 mM imidazole, 1 mM DTT, 0.01 mM EDTA, and 10% glycerol) supplemented with 0.05 mg/ml lysozyme and 1 mM PMSF.

Cells were lysed by sonication and then supplemented with 250 U of universal nuclease (Pierce). The insoluble fraction was separated by centrifugation at 20,000g for 30 min. The supernatant was then incubated with 1 ml of packed Ni-NTA resin (Qiagen) and incubated at 4 °C with gentle agitation for 30 min. The resin was pelleted by centrifugation at 5000g for 5 min, the supernatant was decanted, the resin was resuspended in 5 ml of lysis buffer and loaded onto a gravity column where it was washed with 15 CV of lysis buffer. The resin was further washed with five column volumes of wash buffer (20 mM Tris pH 7.5, 200 mM potassium acetate, 30 mM imidazole, 1 mM DTT, 0.01 mM EDTA, and 10% glycerol). Protein was eluted with five CV of elution buffer (20 mM Tris pH 7.5, 200 mM potassium acetate, 250 mM imidazole, 1 mM DTT, 0.01 mM EDTA, and 10% glycerol). Elution was concentrated in a 30 kDa MWCO Thermo Ultra centrifugal filter (Pierce) and then dialyzed at 1:100 volume to volume into dialysis buffer (20 mM Tris pH 7.5, 200 mM NaCl, and 1 mM DTT) for 16 h. Protein concentrations were determined by A_280_ using a calculated extinction coefficient (Expasy).

### Membrane protein purification

#### SUMO TMD substrates

The main substrate (SUMO-Sec22 TMD) is in a pET28a plasmid backbone. Cloning of all synthetic TMD substrates was conducted *via* traditional PCR/restriction enzyme “cut and paste” methods to swap out Sec22 TMD and verified by Sanger sequencing. The layout of all constructs is His_6_-3C protease site-3xFlag-SUMO-*TMD*-thrombin protease site-HiBiT tag. The DHFR-Sec22 substrate has the same set up except the SUMO domain is replaced with mouse DHFR. Expression and purification of the DHFR-Sec22 substrate was the same as the SUMO-Sec22 substrate.

After transforming the SUMO-TMD plasmid into *E. coli* BL21(DE3)/pRIL, the cells were grown in terrific broth at 37 °C until an *A*_600_ of 0.6 to 0.8, and then induced with 0.25 mM IPTG and grown at RT for an additional 3 to 4 h. Cells were harvested by centrifugation, and resuspended in SUMO-TMD lysis buffer (50 mM Tris pH 7.5, 300 mM NaCl, 10 mM MgCl_2_, 10 mM imidazole, and 10% glycerol) supplemented with 0.05 mg/ml lysozyme (Sigma-Aldrich), 1 mM PMSF and 500 U of benzonase (Sigma-Aldrich), and lysed by sonication. Membrane proteins were solubilized by addition of n-dodecyl-β-D-maltoside (DDM) to a final concentration of 1% and rocked at 4 °C for 30 min. Lysate was cleared by centrifugation at 4 °C for 1 h at 35,000g and purified by Ni-NTA affinity chromatography.

Ni-NTA resin was washed with 10 CV of SUMO-TMD wash buffer 1 (50 mM Tris pH 7.5, 500 mM NaCl, 10 mM MgCl_2_, 10 mM imidazole, 5 mM β-mercaptoethanol (BME), 10% glycerol, and 0.1% DDM). Resin was then washed with 10 CV of SUMO-TMD wash buffer 2 (same as wash buffer 1 except with 300 mM NaCl and 25 mM imidazole) and 10 CV of SUMO-TMD wash buffer 3 (same as wash buffer 1 with 150 mM NaCl and 50 mM imidazole) and then eluted with 3 CV of SUMO-TMD elution buffer (same as wash buffer 3 except with 250 mM imidazole).

The protein was further purified by SEC (Superdex 200 Increase 10/300 GL, GE Healthcare) in 50 mM Tris pH 7.5, 150 mM NaCl, 10 mM MgCl_2_, 5 mM BME, 10% glycerol, and 0.1% DDM. Peak fractions were pooled, aliquots were flash-frozen in liquid nitrogen and stored at −80 °C. Protein concentrations were determined by A_280_ using a calculated extinction coefficient (Expasy).

#### SUMO-SUMO-Sec22

After transforming the SUMO-TMD plasmid into *E. coli* LOBSTR/pRIL, the cells were grown in terrific broth at 37 °C until an *A*_600_ of 0.6 to 0.8, and then induced with 0.25 mM IPTG and grown at 16 °C for an additional 16 h. Cells were harvested by centrifugation, and resuspended in lysis buffer (50 mM Tris pH 7.5, 300 mM NaCl, 10 mM MgCl_2_, 25 mM imidazole, and 10% glycerol) supplemented with 0.05 mg/ml lysozyme (Sigma-Aldrich), 1 mM PMSF and 500 U of benzonase (Sigma-Aldrich), and lysed by sonication. Membrane proteins were solubilized by addition of n-dodecyl-β-D-maltoside (DDM) to a final concentration of 1% and rocked at 4 °C for 30'. Lysate was cleared by centrifugation for at 4 °C for 1 h at 35,000g and purified by Ni-NTA affinity chromatography.

Ni-NTA resin was washed with five CV of SUMO-TMD wash buffer 1 (50 mM Tris pH 7.5, 500 mM NaCl, 10 mM MgCl_2_, 25 mM imidazole, 5 mM BME, 10% glycerol, and 0.1% DDM). Resin was then washed with five CV of SUMO-TMD wash buffer 2 (same as wash buffer 1 except with 300 mM NaCl and 40 mM imidazole) and 10 CV of SUMO-TMD wash buffer 3 (same as wash buffer 1 with 150 mM NaCl and 50 mM imidazole) and then eluted with 3 CV of SUMO-TMD elution buffer (same as wash buffer 3 except with 250 mM imidazole).

The protein was further purified by SEC (Superdex 200 Increase 10/300 Gl, GE Healthcare) in 50 mM Tris pH 7.5, 150 mM NaCl, 10 mM MgCl_2_, 5 mM BME, 10% glycerol, and 0.1% DDM. Peak fractions were pooled, aliquots were flash-frozen in liquid nitrogen and stored at −80 °C. Protein concentrations were determined by *A*_280_ using a calculated extinction coefficient (Expasy).

### Liposome preparation

Liposomes mimicking the lipid composition of the yeast OMM, as well as the thickness and fluidity series, were prepared as described previously (27). Briefly, a lipid film was prepared by mixing chloroform stocks of lipids applicable to each type of liposome.

#### Standard nickel liposomes

Chicken egg phosphatidyl choline (Avanti 840051C), chicken egg phosphatidyl ethanolamine (Avanti 840021C), bovine liver phosphatidyl inositol (Avanti 840042C), synthetic DOPS (Avanti 840035C), synthetic TOCL (Avanti 710335C), and 1,2-dioleoyl-sn-glycero-3-[N-((5-amino-1-carboxypentyl)iminodiacetic acid)succinyl] Nickel salt (Avanti 790404) at a 48:28:10:8:4:2 M ratio with 1 mg of DTT. This resulted in a final concentration of DTT of 8.2 mM for the standard liposomes and 6.3 mM for the T and F series liposomes.

#### T1

Synthetic 14:1, 14:1 phosphatidyl choline (Avanti 850346C) and synthetic 18:1, 18:1 1,2-dioleoyl-*sn*-glycero-3-[*N*-((5-amino-1-carboxypentyl)iminodiacetic acid)succinyl] Nickel salt (Avanti 790404) at a 98:2 M ratio with 1 mg of DTT.

#### T2

Synthetic 16:1, 16:1 phosphatidyl choline (Avanti 850358C) and synthetic 18:1, 18:1 1,2-dioleoyl-sn-glycero-3-[N-((5-amino-1-carboxypentyl)iminodiacetic acid)succinyl] Nickel salt (Avanti 790404) at a 98:2 M ratio with 1 mg of DTT.

#### T3

Synthetic 18:1, 18:1 phosphatidyl choline (Avanti 850375C) and synthetic 18:1, 18:1 1,2-dioleoyl-*sn*-glycero-3-[*N*-((5-amino-1-carboxypentyl)iminodiacetic acid)succinyl] Nickel salt (Avanti 790404) at a 98:2 M ratio with 1 mg of DTT.

#### T4

Synthetic 20:1, 20:1 phosphatidyl choline (Avanti 850396C) and synthetic 18:1, 18:1 1,2-dioleoyl-*sn*-glycero-3-[*N*-((5-amino-1-carboxypentyl)iminodiacetic acid)succinyl] Nickel salt (Avanti 790404) at a 98:2 M ratio with 1 mg of DTT.

#### T5

Synthetic 22:1, 22:1 phosphatidyl choline (Avanti 850398C) and synthetic 18:1, 18:1 1,2-dioleoyl-*sn*-glycero-3-[*N*-((5-amino-1-carboxypentyl)iminodiacetic acid)succinyl] Nickel salt (Avanti 790404) at a 98:2 M ratio with 1 mg of DTT.

#### F1

Synthetic 18:1, 18:1, *trans* phosphatidyl choline (Avanti 850376C) and synthetic 18:1, 18:1 1,2-dioleoyl-*sn*-glycero-3-[*N*-((5-amino-1-carboxypentyl)iminodiacetic acid)succinyl] Nickel salt (Avanti 790404) at a 98:2 M ratio with 1 mg of DTT.

#### F2

Synthetic 18:0, 18:1 phosphatidyl choline (Avanti 850467C) and synthetic 18:1, 18:1 1,2-dioleoyl-*sn*-glycero-3-[*N*-((5-amino-1-carboxypentyl)iminodiacetic acid)succinyl] Nickel salt (Avanti 790404) at a 98:2 M ratio with 1 mg of DTT.

#### F3

Synthetic 18:1, 18:1, *cis* phosphatidyl choline (Avanti 850375C) and synthetic 18:1, 18:1 1,2-dioleoyl-*sn*-glycero-3-[*N*-((5-amino-1-carboxypentyl)iminodiacetic acid)succinyl] Nickel salt (Avanti 790404) at a 98:2 M ratio with 1 mg of DTT.

#### F4

Synthetic 18:2, 18:2 phosphatidyl choline (Avanti 850385C) and synthetic 18:1, 18:1 1,2-dioleoyl-*sn*-glycero-3-[*N*-((5-amino-1-carboxypentyl)iminodiacetic acid)succinyl] Nickel salt (Avanti 790404) at a 98:2 M ratio with 1 mg of DTT.

Chloroform was evaporated under a gentle steam of nitrogen and then left on a vacuum (<1 mTorr) overnight. Lipid film was fully resuspended in liposome buffer (50 mM Hepes KOH pH 7.5, 15% glycerol, and 1 mM DTT) to a final concentration of 20 mg/ml and then subjected to five rapid freeze-thaw cycles with liquid nitrogen. Liposomes were extruded 15 times through a 100 nm filter at 60 °C, distributed into single-use aliquots, and flash-frozen in liquid nitrogen.

### Reconstitution into liposomes

For extraction assays, proteoliposomes were prepared by mixing 2.5 μM TA protein (SUMO-TMD), and 2 mg/ml of nickel liposomes in reconstitution buffer. Detergent was removed by adding 25 mg of biobeads (Bio-Rad #1523920) and rotating the samples for 16 h at 4 °C. Material was removed from biobeads and added to fresh 25 mg biobeads and rotated for another 1 to 3 h at RT. Unincorporated TA protein was precleared by incubating the reconstituted material with excess (5 μM) GST-SGTA and GST-calmodulin and passing over a glutathione spin column (Pierce #16103); the flow through was collected and used immediately for dislocation assays.

### C-laurdan spectroscopy

C-laurdan spectroscopy was performed using 10 μl of preformed F-series liposomes and 90 μl of extraction buffer (50 mM Hepes pH 7.5, 200 mM KAc, 7 mM MgAc, 2 mM DTT, and 10 μm Ca^2+^) to match overnight reconstitution volume. Final 50 μg/ml of C-laurdan dye (Thermo Fisher Scientific) was added, and a fluorescence spectrum was read. The sample was excited at 375 nm, and an emission spectrum from 400 to 600 nm with 3 nm step size was recorded. The GP value (−1 most disordered, +1 most ordered membranes) was measured as described previously (72) by integrating the intensities between 400 and 460 nm (*I*_Ch1_), and 470 and 530 nm (*I*_Ch2_):

$$\text{GP}=\left(I_{\text{CH}1}-I_{\text{CH}2}\right)\;/\;\left(I_{\text{CH}1}+I_{\text{CH}2}\right)\text{.}$$

### Extraction assay

Extraction assays contained 28.6 μl of precleared proteoliposomes, 5 μM GST-SGTA, 5 μM GST-calmodulin, 3 μM Msp1 hexamer, 8 μM MBP-Ubiquitin, 1 mg/ml bovine serum albumin (Sigma-Aldrich), and 80 mM ATP and the final volume was adjusted to 105 μl with extraction buffer (50 mM Hepes KOH pH 7.5, 200 mM potassium acetate, 7 mM magnesium acetate, 2 mM DTT, and 0.1 μM calcium chloride). Samples were incubated at 30 °C for 30 min and then transferred to ice. Samples were transferred to polycarbonate centrifuge tubes (Beckman-Coulter #343776) and centrifuged at 150,000*g* in TLA-120.1 Fixed-Angle Rotor (Beckman-Coulter) for 30′ at 4 ˚C. The top 20 μl was collected and combined with 1.5 μM LgBiT and the final volume was adjusted to 50 μl with extraction buffer. After incubating at RT for 25 min, samples were plated onto white 96-well plates (Corning #07201204) and 20 μl of lytic furimazine (Promega #N3030) was added. Immediately plates were read at 470 nm wavelength to measure luminescence.

Extraction assays with DHFR-Sec22 were performed as described above except DMSO or methotrexate were added 5 min prior to initiation of the assay with ATP. A 1 mM methotrexate stock solution was prepared in DMSO and then added to at a final concentration of 5 μM. The final concentration of DMSO in the assay was 0.5% of the reaction volume.

### Standard curve measurements

Between 4 and6 dilutions of precleared liposomes used in the extraction assay were made. Dilutions ranged from 5% to 30% of the total amount of precleared liposomes used in the extraction assay and were brought up to 105 μl with extraction buffer (50 mM Hepes KOH pH 7.5, 200 mM potassium acetate, 7 mM magnesium acetate, 2 mM DTT, and 0.1 μM calcium chloride). This was then diluted to make 15%, 10%, and 5% samples. Twenty microliters from each was then combined with 1.5 μM LgBiT, and the final volume was adjusted to 50 μl with extraction buffer. After incubating at RT for 25 min, samples were plated onto white 96-well plates (Corning #07201204), and 20 μl of lytic furimazine (Promega #N3030) was added. Plates were immediately read at 470 nm wavelength to measure luminescence.

### Protease protection assay

Protease protection assay was carried out in a 10 μl total volume with 7 μl of precleared liposomes, 2 U of thrombin protease, and 1% of Triton X-100 (where indicated). Samples were incubated at RT for 1 h and then 1 μl of 200 mM PMSF was added. The samples were reverse quenched into 90 μl of boiling 1% SDS and incubated at 95 ˚C for 10 min. Anti-HiBiT western blot was performed with 1:5000 dilution of mouse anti-HiBiT (Promega, RRID: AB_3665694) and 1:10,000 dilution of goat anti-mouse-HRP secondary antibody (Invitrogen, RRID: AB_228307). Anti-HiBiT antibody was validated by western blot with recombinantly purified protein containing the HiBiT tag. Western blots were developed with SuperSignal West Femto Maximum Sensitivity Substrate (Thermo Fisher Scientific) and imaged on a ChemiDoc imager (Bio-Rad).

### Statistical analysis

Statistical analysis was performed in Graphpad Prism. Comparisons between two samples used an unpaired, two-tailed *t* test. Comparisons between more than two samples used 1-way ANOVA with Dunnett's *post hoc* test.

## Data availability

Data are available upon request.

## Supporting information

This article contains supporting information (28, 29).

## Conflict of interest

The authors declare that they have no conflicts of interest with the contents of this article.
