# Supplementary material for: Lipid bilayer properties govern substrate engagement and extraction by the AAA+ ATPase Msp1
Source: J Biol Chem. 2025 Aug 19;301(10):110614. doi: 10.1016/j.jbc.2025.110614 (PMC12624790; doi:10.1016/j.jbc.2025.110614)
Supplement: Figure S1 [file mmc1.docx]

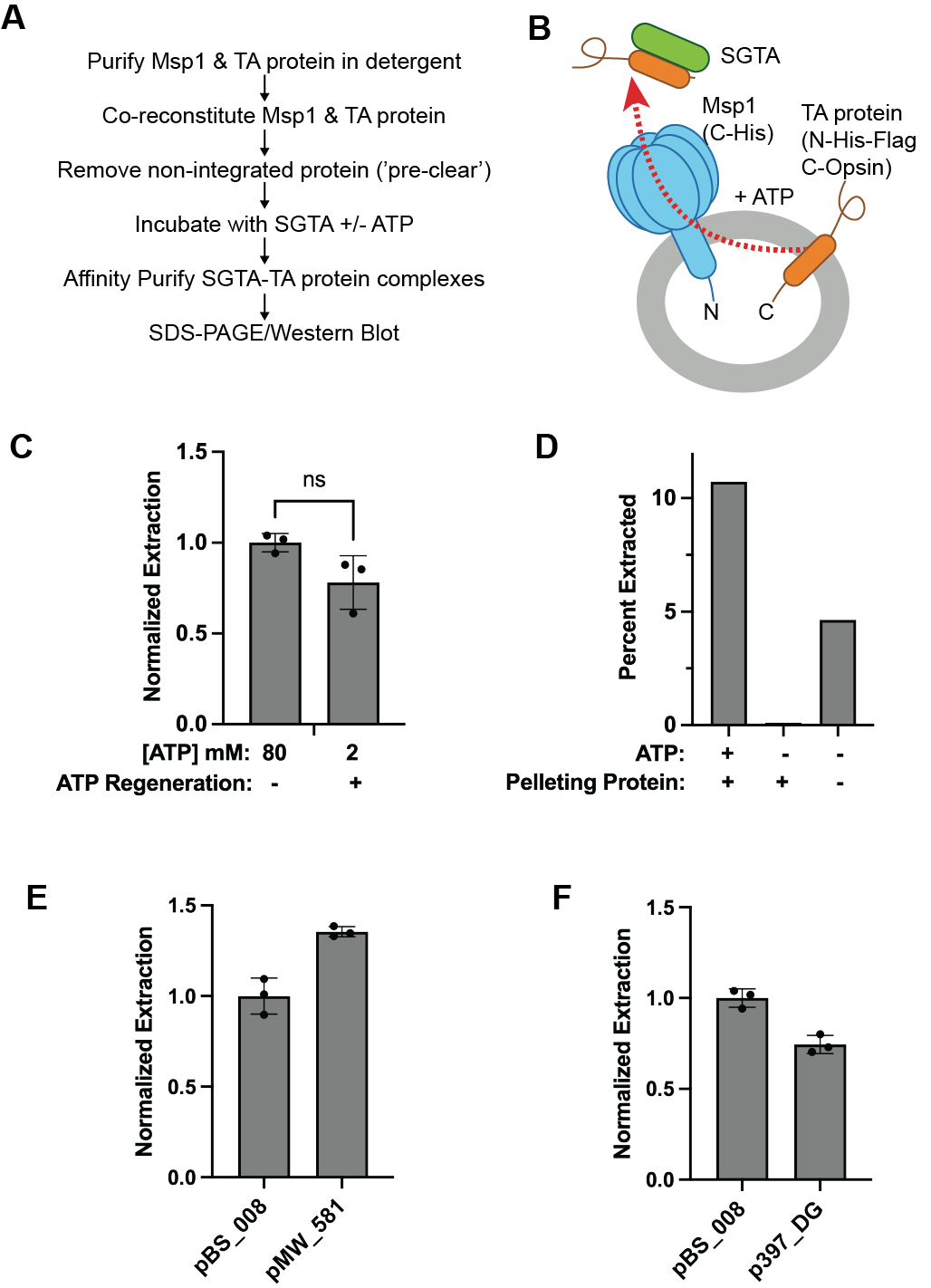


**Figure S1: Validation of the split-luciferase based extraction assay**

1. Workflow for previously developed extraction assay, which relies on immunoprecipitation of the chaperone SGTA and western blot of the substrate.
2. Diagram for previously developed extraction assay
3. Effect of ATP concentration on extraction activity. Extraction assay with the standard SUMO-Sec22 model substrate in standard liposomes. Extraction activity was normalized such that 80 mM ATP is 1.0. Error bars show standard deviation from 3 replicates from two separate reconstitutions. Difference in means is not significant by unpaired, 2-tailed t-test.
4. Addition of an inert His_6_-tagged protein pBS_008 prior to ultracentrifugation improves liposome pelleting efficiency and reduces background activity.
5. Changing the inert protein used in pelleting has a modest effect on overall extraction levels. Extraction assay with the standard SUMO-Sec22 model substrate in standard liposomes. Extraction activity was normalized such that pelleting with pBS_008, which is used throughout the rest of the paper, is 1.0. Extraction with pBS_008 and pMW_581 were performed in parallel using the same preparations of substrates, chaperones, liposomes, and Msp1. Error bars show standard deviation from 3 replicates from two separate reconstitutions.
6. Changing the inert protein used in pelleting has a modest effect on overall extraction levels. Extraction assay with the standard SUMO-Sec22 model substrate in standard liposomes. Extraction activity was normalized such that pelleting with pBS_008, which is used throughout the rest of the paper, is 1.0. Extraction with pBS_008 and p397_DG were performed in parallel using the same preparations of substrates, chaperones, liposomes, and Msp1. Error bars show standard deviation from 3 replicates from two separate reconstitutions.
